# Facial Cellulitis Mimicking Ludwig’s Angina in a Patient with Chronic Myelogenous Leukemia on Dasatinib Therapy

**DOI:** 10.3390/idr18040075

**Published:** 2026-07-15

**Authors:** Nicole Liang, Jenna DeTemple, Christopher E. Potts, Usman Alizai, Charles Meadows

**Affiliations:** 1 Joan C. Edwards School of Medicine, Marshall University, Huntington, WV 25701, USA; liangn@marshall.edu (N.L.); detemple1@marshall.edu (J.D.); cmeadows@marshall.edu (C.M.III); 2 Department of Surgery, The Ohio State University Wexner Medical Center, Columbus, OH 43210, USA; usman.alizai@osumc.edu

**Keywords:** facial cellulitis, Ludwig’s angina, odontogenic infection, immunosuppression, dasatinib, chronic myelogenous leukemia

## Abstract

Background: Immunosuppressed patients are at an increased risk for developing odontogenic and orofacial infections, which can present with atypical processes and features that may mimic rare but life-threatening infections such as Ludwig’s angina. Differentiating cellulitis from a deep neck space infection is often challenging in this population in acute settings due to a broad differential diagnosis and blunted inflammatory responses. This diagnostic uncertainty complicates acute risk stratification and may delay recognition of conditions requiring early airway evaluation and intervention. Case Presentation: We present the case of a 28-year-old male with chronic myelogenous leukemia on immunosuppression with dasatinib who developed unilateral facial swelling and severe odontogenic pain that was refractory to empiric antibiotic therapy. The patient’s presentation with rapid clinical progression, early trismus, submandibular involvement, and floor-of-mouth tenderness raised significant concern for evolving Ludwig’s angina. Laboratory evaluation demonstrated elevated inflammatory markers, including erythrocyte sedimentation rate and C-reactive protein, further complicating early assessment. Imaging was promptly obtained to determine the nature of the infection, and the patient was admitted for intravenous antibiotic therapy and airway monitoring. Clinical improvement ensued. Conclusions: This case highlights the diagnostic overlap between facial cellulitis and Ludwig’s angina and underscores the importance of prompt imaging, airway monitoring, and clinical vigilance for risk stratification of immunocompromised patients in the acute setting to prevent life-threatening complications.

## 1. Introduction

Odontogenic infections are often encountered in clinical practice and represent a significant disease burden globally. They are the most common cause of deep neck space infections in adults, accounting for 48–78% of cases [1]. They are usually polymicrobial, with alpha-hemolytic streptococci most often isolated [2]. Most odontogenic infections arise from dental caries or periodontal disease and remain localized, presenting with worsening dental pain and localized swelling [3]. However, untreated cases may spread further. The fascial planes of the face and neck facilitate rapid bacterial dissemination, resulting in complications such as facial cellulitis, abscess formation, osteomyelitis, or Ludwig’s angina. Disease progression depends on several factors, including the patient’s previous oral health, the virulence of the pathogen, adequacy of source control, and timely treatment initiation. Consequently, prompt recognition and management of severe odontogenic infections are essential to preventing significant morbidity and mortality [3,4].

Immunosuppressed individuals, particularly those with hematologic malignancies or receiving systemic antineoplastic therapy, are especially vulnerable to severe orofacial disease since impaired host defenses limit the ability to contain local bacterial dissemination. Cancer drugs, including chemotherapies and targeted therapies like tyrosine kinase inhibitors, may further increase susceptibility through treatment-related oral mucositis [5]. Thus, even minor or asymptomatic odontogenic infections can progress beyond the oral cavity to readily spread across fascial planes and cause deep tissue infection. Progression into severe systemic disease is further facilitated by failure to address the offending tooth, antibiotic non-compliance, poor oral hygiene, and delayed medical evaluation, among other factors [3,4]. Prior to initiation of immunosuppressive antineoplastic therapies, patients should undergo a thorough evaluation for active odontogenic, sinonasal, and cutaneous infections. Chronic dental disease is particularly important because it can serve as a persistent bacterial reservoir capable of progression to deep neck space infections when host immunity is compromised. Treatment of active odontogenic disease should be completed 2 weeks before treatment initiation [6].

Dasatinib, a BCR-ABL tyrosine kinase inhibitor (TKI), is used in the treatment of blood cancers such as chronic myelogenous leukemia (CML). Reported oral adverse effects of the drug include oral soft tissue injury or mucosal inflammation, leading to mucositis and stomatitis in 1–10% of patients [7]. The mechanism of dasatinib that causes these effects is not fully elucidated, but Src kinase inhibition and disruption of epithelial integrity may play roles. Dasatinib may also impair neutrophil and lymphocyte function, potentially increasing susceptibility to atypical or more severe infections [8]. These adverse effects, when combined with poor oral hygiene or tobacco use, only exacerbate the infections [5]. As a result, clinical presentation of odontogenic infections can be complicated and contribute to diagnostic uncertainty in immunocompromised patients.

Ludwig’s angina is a rapidly progressive infection of the deep spaces of the neck and floor of the mouth that may result in sudden airway compromise. While contrast-enhanced CT scans are invaluable for defining the extent of infection and evaluating airway patency, Ludwig’s angina remains a largely clinical diagnosis [9]. Although severe orofacial disease has been described in immunocompromised patients, reports of facial cellulitis clinically appearing as early Ludwig’s angina during dasatinib therapy are limited. We present the case of a 28-year-old male with CML receiving dasatinib and recurrent dental disease whose seemingly routine episode of localized tooth pain rapidly progressed to substantial submandibular swelling with ipsilateral head-and-neck involvement, concerning for a deep neck space infection. This case highlights the diagnostic uncertainty between early Ludwig’s angina and facial cellulitis in immunocompromised patients, demonstrating the importance of clinical vigilance, early imaging, and prompt management for severe odontogenic infections.

## 2. Case Presentation

A 28-year-old male with a history of CML, Chiari malformation, and longstanding dental neglect presented to the emergency department with significant unilateral facial swelling, severe odontogenic pain at rest, and increasing difficulty with opening the mouth. Three days prior, the patient was evaluated for a dental infection and treated with oral penicillin VK. Despite compliance with medication dosing instructions, symptoms had rapidly progressed with increasing jaw swelling, pain radiating to the left ear and anterior neck, and pain with mastication, consistent with potential early trismus. The patient also noted clear purulent discharge from the left ear and reported fever and chills. This abrupt worsening of swelling refractory to early antibiotic therapy over several days raised suspicion for an evolving deep neck space infection.

On arrival, the patient reported increasing difficulty with opening the mouth, inability to masticate, and floor-of-mouth tenderness resulting in decreased oral intake. The left lower face and jaw were swollen and erythematous. Patient vital signs included a temperature of 97.5 °F, HR of 67 beats/min, BP of 164/101 mmHg, RR of 16 breaths/min, and oxygen saturation of 97% on room air. A repeat blood pressure reading six minutes later was 153/90 mmHg. On examination, significant pain was detected around tooth #15 and around the left upper and lower molars with firm induration tracking along the mandible toward the submandibular space. The patient rated the pain as an 8 on a 1–10 scale. Mild elevation tenderness of the floor of the mouth was appreciated. Pain of the left pinna and tragus was detected with palpation along with tender cervical lymphadenopathy. Intraoral examination showed extensive dental caries, consistent with a likely odontogenic source.

The patient was admitted to the hospital for medical management and started on intravenous antibiotic infusions, including piperacillin/tazobactam and vancomycin. Ketorolac tromethamine and morphine were administered for pain control. Blood pressure subsequently decreased to 114/88. Other vital signs were normal. White blood cell count was 8.40, and the remainder of the CBC and CMPs were within reference ranges. ESR and CRP were elevated at 17 mm/h and 8.36 mg/dL, respectively (Figure A1). Advanced symptomatic workup determined the presence of facial cellulitis without abscess or gas inclusions (Figure 1). Peripheral blood cultures taken at 24, 48, 72, and 96 h after admission remained negative, so the original empiric therapy was continued throughout his hospital stay. The patient insisted on being discharged prior to complete eradication of the infection and was discharged with a 7-day course of oral doxycycline and clindamycin to provide coverage for both streptococcus and staphylococcus organisms. Appointments were made for the patient to seek outpatient dental care to remove tooth #15 and address his other dental caries. After discharge, the patient was lost to follow-up, so it is unknown whether source control was ever gained.

Although the patient denied drooling or dyspnea, the constellation of rapid symptom progression, immunocompromised treatment status, submandibular involvement, sublingual discomfort, and pain with mouth opening raised alternate concerns for early Ludwig’s angina, necessitating urgent diagnostic evaluation and complete workup before discharge. While the airway was not compromised on arrival, the pattern of swelling and symptomology was especially concerning for the presence of a deep neck space infection.

Informed consent was obtained from the patient regarding the use of clinical findings, laboratory results, and imaging studies for research purposes. The patient agreed to all the above. After discussion with our institution’s medical IRB, it was determined that IRB approval was not required for the publication of this case report.

## 3. Discussion

Our patient presented with severe submandibular pain and swelling from a progressive odontogenic infection refractory to standard antibiotic treatment, eventually being diagnosed with facial cellulitis. The differential diagnosis for mandibular swelling in an immunocompromised individual is extensive. The most common cause is odontogenic infection, with immunosuppression increasing the risk of rapid progression and deep neck space involvement [10]. Other etiologies, such as facial cellulitis [11], submandibular sialadenitis, parotitis [12], reactive cervical lymphadenopathy [13], and mandibular osteomyelitis [14], also present with similar facial pain and swelling. Given this patient’s acute onset, focal pain and associated jaw tenderness, and history of recurrent dental caries, the initial diagnosis of dental abscess was likely [10]. However, the patient’s subsequent clinical deterioration despite empirical treatment with penicillin VK raised significant concern for Ludwig’s angina, a potentially life-threatening condition requiring immediate intervention. Since delayed recognition of deep neck space infections can result in sudden airway compromise, early management was warranted despite no signs of acute respiratory distress.

Distinguishing facial cellulitis from early Ludwig’s angina is a diagnostic challenge due to a significant overlap of nonspecific symptoms. Both Ludwig’s angina and facial cellulitis most often originate from odontogenic infection. Both present with localized pain, swelling, erythema, and tenderness, as well as systemic symptoms such as fevers and malaise in some cases. Causative pathogens are similar as well, most commonly beta-hemolytic streptococci, staphylococci, and anaerobic oral flora [9,11]. However, the pathophysiology of these diseases differs in anatomical location, spread, and airway involvement. Ludwig’s angina is a rapidly progressing cellulitis of the submandibular, sublingual, and submental space; the direct anatomical continuity of these deep fascial planes facilitates bilateral involvement. Ludwig’s angina causes diffuse hardened indurations and edema, leading to elevation and posterior displacement of the tongue. As such, airway obstruction is a life-threatening complication [8]. In contrast, facial cellulitis is a diffuse unilateral infection of the dermis and subcutaneous tissue. Spread is through superficial lymphatic channels, so facial cellulitis does not involve the floor of the mouth or cause airway compromise. While these infections can be severe, the immediate threat of mortality is not present [11]. In immunocompromised patients, the delineation between the two becomes murky. Ludwig’s angina in its early stages can have a more insidious progression, with its classic findings of bilateral “woody” submandibular induration and tongue elevation emerging later. This pattern is more likely in patients with atypical immune responses [15,16,17]. Conversely, facial cellulitis caused by aggressive organisms, especially in immunocompromised patients, may spread along fascial planes and produce patterns of edema that suggest deep space or submandibular involvement [11,17]. Thus, differentiation between these two pathologies is crucial for appropriate management.

Our patient’s presentation illustrates these atypical findings. His rapidly progressive unilateral facial swelling, submandibular tenderness, floor-of-mouth pain, and potential trismus were concerning for Ludwig’s angina. However, the patient did not present with tongue elevation, woody indurations, or respiratory compromise. Despite not fitting a single clinical picture, it was prudent to treat his condition as a case of early Ludwig’s angina. Thus, an inpatient hospital admission and early advanced imaging were pursued. Interestingly, the patient’s CBCs and BMPs remained unremarkable throughout his encounter in the ER and subsequent hospital stay, despite an elevated ESR and CRP indicating inflammation. The patient also did not present with a fever even with a clinically significant infection. While these findings can be seen in immunocompromised patients who fail to mount a leukocytic response, the lab value discordance further complicated early assessment. Therefore, clinicians cannot solely rely on a normal CBC to evaluate severe odontogenic infections in patients undergoing immunomodulatory therapy.

The patient’s history of CML with long-term dasatinib treatment likely contributed to an atypical clinical presentation. While dasatinib treatment may restore some immune function in CML patients, the drug is an immunosuppressant [18]. Major side effects include myelosuppression and neutropenia, increasing infection risk even compared to other TKIs [8]. Functional immunosuppression can persist even after white cell counts recover [19]. Dasatinib also inhibits the proinflammatory functions of neutrophils, decreases the proliferation and activation of T-cells and B-cells, and reduces interferon- and granzyme B secretion [20,21,22]. All these alterations reduce the host’s ability to recognize and contain soft tissue infections, predisposing to more diffuse patterns of inflammatory spread. Combined with a history of recurrent dental infections and extensive prior antibiotic exposure (increasing the risk of antibiotic-resistant oral flora), our patient was uniquely vulnerable to developing a complicated infectious course. Leukemia patients are at a higher risk of developing oral infections, with occasional progression to facial cellulitis or Ludwig’s angina if not properly treated [23]. TKI-related mucosal barrier injury further increases susceptibility to bacterial invasion [24]. However, facial cellulitis mimicking early Ludwig’s angina after dasatinib therapy is poorly characterized. While a causal relationship cannot be established, this presentation highlights how targeted therapies may be associated with atypical presentations of rapidly progressive odontogenic infections. Since typical clinical and laboratory findings may be obscured in this population, physicians should maintain a higher degree of suspicion for life-threatening complications and adjust management according to the overall clinical picture rather than isolated findings.

These findings highlight the importance of screening for active odontogenic infections prior to initiating immunosuppressive antineoplastic therapies. Existing periodontal disease or untreated dental abscesses may serve as persistent bacterial reservoirs that can progress to significant infections once host immunity decreases. During therapy, new dental pain, facial swelling, or other signs of oral infection should prompt early evaluation and treatment. Definitive source control through timely dental intervention remains essential [6].

Prompt advanced imaging is beneficial for diagnostic clarification. Differentiating between Ludwig’s angina and facial cellulitis most often utilizes contrast-enhanced CT. This imaging modality can reliably assess deep neck space involvement, the extent of infection, drainable abscesses, and risk of airway compromise. Ludwig’s angina presents as bilateral inflammatory involvement of the submandibular, sublingual, and submental spaces with diffuse soft tissue edema and possible airway compromise. Abscess or gas formation within deep neck tissue may occur in more advanced disease. Facial cellulitis is seen as localized soft tissue edema and inflammatory fat stranding without involvement of the deep neck spaces or a drainable abscess [8,10]. However, imaging cannot replace a thorough clinical assessment. Ludwig’s angina is still primarily a clinical diagnosis, especially since early disease may not show the classic radiographic findings. MRIs may be beneficial to evaluate intracranial/skull involvement or detect abscesses or osteomyelitis but are generally reserved for clinically stable patients [25]. Point-of-care ultrasound can be used if CT is unavailable or contraindicated [26]. In this case, CT imaging showed no abscesses or gas in deep neck spaces, making severe facial cellulitis a more likely diagnosis than Ludwig’s angina. This information from imaging supported the use of conservative medical management and prevented unnecessary surgical intervention.

Treatment of facial cellulitis depends on local resistance patterns and prior antibiotic exposure. Broad-spectrum antibiotic regimens should include streptococci, staphylococci, and Gram-negative coverage [27]. For mild cases, amoxicillin–clavulanic acid is typically prescribed, while clindamycin or moxifloxacin are alternatives for beta-lactam allergy [28]. Caution should be used when prescribing fluoroquinolones, given significant adverse effects such as tendinitis/tendon rupture, peripheral neuropathy, and cardiac arrhythmias [27]. Inpatient admission is recommended for more severe cases or immunocompromised patients. IV ampicillin-sulbactam is first-line for moderate to severe facial cellulitis; add metronidazole for anaerobic coverage [29]. In severely immunocompromised patients, use IV vancomycin plus either piperacillin-tazobactam, imipenem, or meropenem, especially if there is previous beta-lactam exposure or potential MRSA colonization [27]. Use blood cultures and/or tissue aspirates to guide antibiotic therapy after initial management. Antibiotics should be given for five days or longer. If deep or necrotizing infection is suspected/visualized, surgical debridement may be necessary [27,28].

Source control of the original odontogenic infection is considered definitive treatment. Incision and drainage of oral abscesses should be done as soon as possible, regardless of other dental work. Extraction of the causative tooth early removes the bacterial reservoir, leading to significantly faster resolution and lower complication rates [6]. Given our patient’s clinical severity and immunocompromised status, empiric IV vancomycin and piperacillin-tazobactam were started upon admission. Due to negative blood cultures, potentially from prior outpatient antibiotic therapy, his treatment regimen was not altered. The patient experienced improvement of pain and swelling over 4 days in the hospital. However, he did not wish to remain admitted, so an oral course of doxycycline (MRSA coverage) and clindamycin was prescribed to clear the remaining infection. It is unknown whether source control was obtained in this patient, as he was referred to outpatient dental clinics for tooth extraction, and no follow-up information after discharge is known.

## 4. Conclusions

The present case illustrates how facial cellulitis can closely resemble early Ludwig’s angina in an immunocompromised patient with a history of periodontal infections. Despite recent antibiotic treatment for dental abscess, the patient failed to improve, necessitating additional investigation into disease etiology. Nonspecific signs and symptoms created significant diagnostic uncertainty. The patient’s presentation and final diagnosis underscore the need for clinical vigilance, early imaging, and a high index of suspicion for antibiotic-resistant organisms when immunocompromised patients fail to respond to standard regimens. Recognition of these atypical presentations is critical for initiating timely management and preventing life-threatening complications.

Key Clinical Learning Points

1. Immunocompromised patients on tyrosine kinase inhibitor therapy may develop severe orofacial disease with an atypical clinical presentation and relatively normal laboratory studies, necessitating a high index of suspicion for life-threatening complications.
2. Ludwig’s angina is largely a clinical diagnosis, but contrast-enhanced CT is invaluable for assessing deep neck space involvement, airway compromise, and abscess formation to guide further management (medical vs. surgical).
3. Progression of infectious symptoms despite appropriate outpatient antibiotics should prompt urgent reassessment for resistant pathogens, insufficient source control, or involvement of deep neck spaces.
4. Early and continued airway evaluation is critical in patients with rapidly progressive submandibular or floor-of-mouth swelling to prevent significant morbidity/mortality, regardless of early imaging findings.
5. Consistent dental examinations and definitive source control are essential to treat odontogenic infections before they spread to other orofacial structures, especially in patients undergoing immunosuppressive antineoplastic regimens.

## Figures and Tables

**Figure 1:**
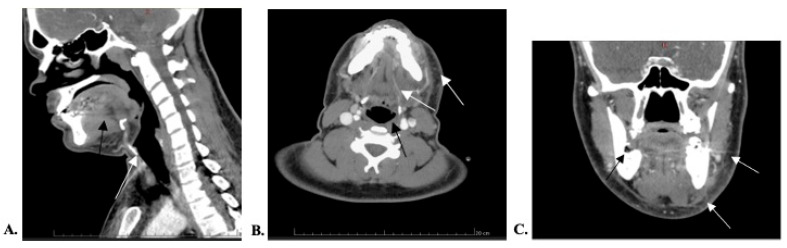
(**A**) Sagittal soft-tissue CT of the patient’s head and neck shows facial cellulitis of the left face with no soft tissue gas or abscess (black arrow) and patent airways (white arrow). (**B**) Coronal soft-tissue CT of the patient’s head shows dental caries (black arrow) and extensive left-sided swelling of the face and jaw (white arrow). (**C**) Axial soft-tissue CT of the patient’s head shows diffuse inflammatory edema of the left submandibular and jaw region with no soft-tissue gas (white arrows). The airway is patent (black arrow).

**Figure A1:**
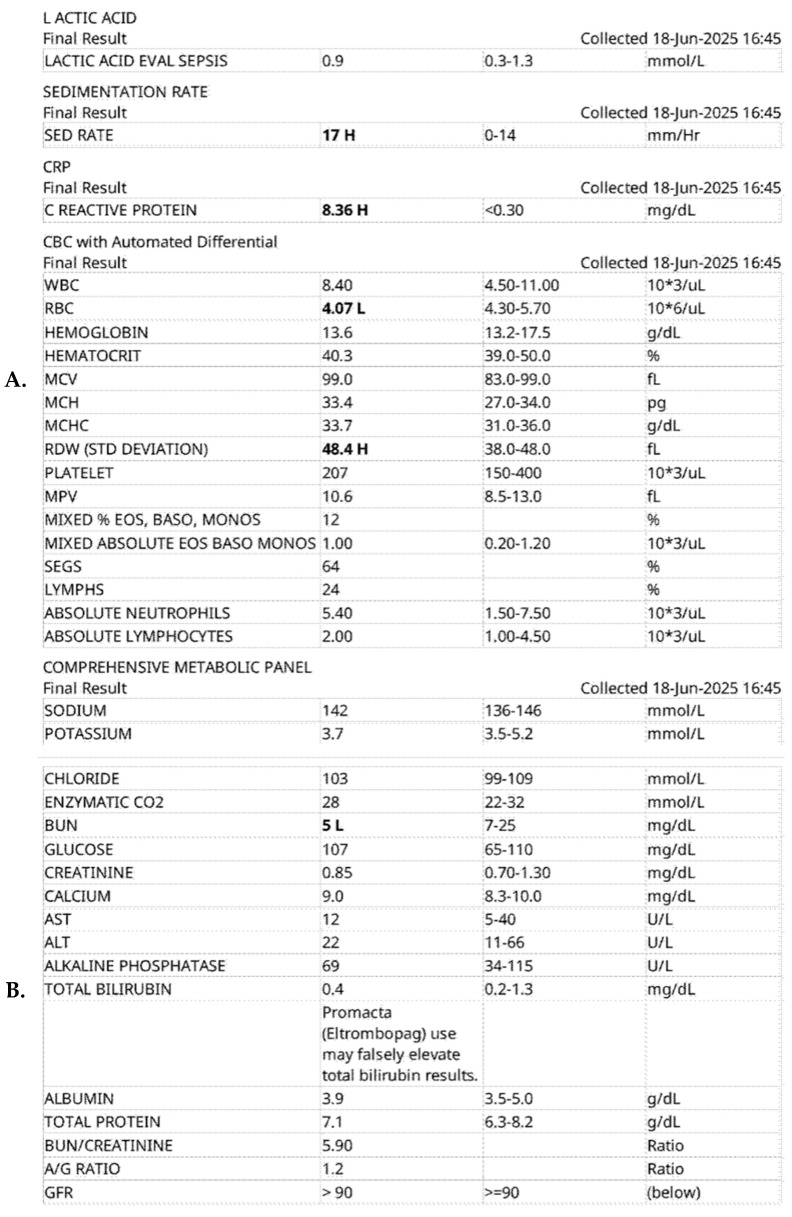

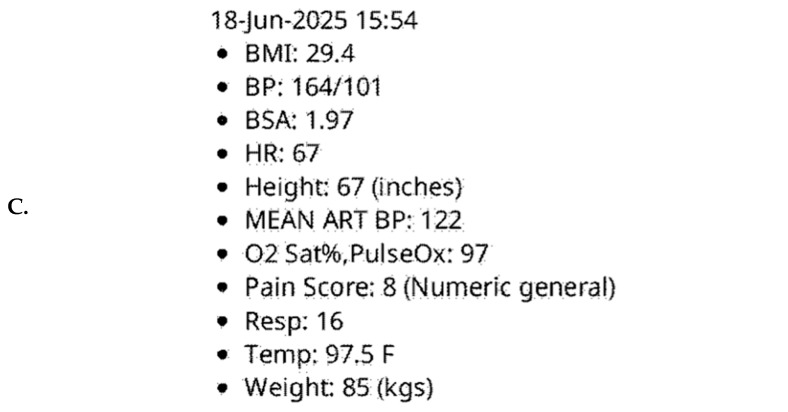
(**A**) Patient’s CBC and partial CMP on admission. (**B**) Patient’s CMP on admission. (**C**) Patient’s vital signs on admission.

## Data Availability

No new data was generated through this report. The data presented in this case report is not publicly available because it contains protected health information that could compromise patient privacy.
